# Influence of E-Cigarette and Cannabis Vaping on Orthodontically Induced Tooth Movement and Periodontal Health in Patients Undergoing Orthodontic Therapy

**DOI:** 10.3390/ijerph19116518

**Published:** 2022-05-27

**Authors:** Dimitrios Michelogiannakis, Irfan Rahman

**Affiliations:** 1 Department of Orthodontics and Dentofacial Orthopedics, Eastman Institute for Oral Health, University of Rochester, New York, NY 14620, USA; 2 Department of Environmental Medicine, University of Rochester Medical Center, Rochester, New York, NY 14642, USA; irfan_rahman@urmc.rochester.edu

The frequency of e-cigarette vaping of nicotine and marijuana products is increasing among adolescents and young adults; the detrimental effects of vaping on general and oral health have not yet been thoroughly defined. It is speculated that nicotine, marijuana/cannabis products including cannabidiol (CBD), and added flavoring chemicals in e-cigarettes may alter the inflammatory response and periodontal tissue remodeling processes during orthodontically induced tooth movement, leading to potential oral side effects in patients undergoing orthodontic therapy such as increased alveolar bone loss and root resorption. The present editorial discusses the state of current evidence regarding the impact of rising vaping on orthodontically induced tooth movement and periodontal health in patients undergoing orthodontic therapy and suggests possible directions for future research.

Tobacco smoking is a leading cause of preventable mortality worldwide and is associated with the development of various diseases, such as cancer, heart, and respiratory diseases. Nicotine is one of the most detrimental toxic compounds of tobacco products/smoke; it causes tissue alterations including impaired angiogenesis, tissue perfusion, and healing as well as altered collagen synthesis and bone remodeling [1,2]. From an orodental aspect, tobacco smoking induces changes in the oral microflora, promoting the colonization of key periodontal pathogens such as *Porphyromonas gingivalis* and *Candida* species [3,4]. Nicotine consumption has also been associated with an increased risk of oral cancer and oral inflammatory and periodontal diseases such as periodontitis [5].

Electronic cigarettes (E-cigarettes) are battery-powered devices containing a tank filled with liquid (which is usually flavored) and they deliver varying degrees of nicotine after heating the liquid into an inhalable aerosol [6]. It is commonly perceived that vaping e-cigarettes is less harmful to health compared with conventional tobacco products [7]. This potentially flawed assumption has led to a rapid emergence of e-cigarette vaping, especially among adolescents and young adults attempting to quit/reduce tobacco smoking. Nonetheless, e-cigarette vaping causes health effects similar to cigarette smoking [8].

In addition to nicotine, e-cigarette liquid also contains propylene glycol (PG), vegetable glycerin (VG) along with traditional (taste of tobacco, menthol, or mint) and nontraditional flavoring chemicals (with tastes such as candy and fruit), which are not found and are even prohibited in combustible cigarettes [9,10]. The use of nontraditional flavors in e-cigarettes has increased the frequency of vaping among youth, and it has been positively associated with vaping continuation in adolescents [9]. There are over 8000 different flavors/chemicals used in e-cigarettes with unknown and potentially deleterious health effects [11]. For instance, vanillin, menthol, and ethyl maltol (which can be found in major e-cigarette brands) have been associated with cytotoxic effects, the induction of oxidative stress and inflammation, and DNA damage in lung cells [10]. Moreover, various toxicity and inflammatory biomarkers have been found to be elevated in e-cigarette vapers compared to never-smokers in biofluids including serum, urine, and saliva [12]. Furthermore, e-cigarette-derived nicotine and flavoring chemicals have several effects on cellular and mitochondrial function, including the inhibition of myofibroblast differentiation, which may in turn lead to impaired wound healing and gel contraction in lung tissues [13,14].

Flavored e-cigarettes have also been shown to cause alterations in oral tissues including increased oxidative and inflammatory responses in human periodontal ligament fibroblasts and lead to a state of irreversible growth arrest in the oral epithelium [15]. In this regard, e-cigarette vaping increases oral inflammation [16], and it may increase the risk of periodontal tissue destruction, oral submucous fibrosis, and oral cancer [17]. Nicotine binds to its nicotinic receptors (alpha3 and alpha7 nicotinic acetylcholine receptors (AChRs)) and flavor aldehydes potentially with TRP receptors. Similarly, it is perceived that synthetic nicotine and their isomers will have the same effects [18,19]. Further a recent emergence of synthetic cooling agents along with nicotine will aggravate the toxicities [15,20,21,22,23,24,25].

Marijuana (Cannabis sativa) contains over 100 cannabinoids with the main compounds being the psychoactive synthetic delta-9-tetrahydrocannabinol (THC) and non-psychoactive cannabidiol (CBD) compound [26]. Vaping cannabis (THC or CBD) among adolescents and adults is rising, particularly in the U.S. where the legalization of recreational cannabis use has occurred in several states [27,28]. Moreover, it has been reported that Cannabis use has increased during the COVID-19 pandemic [29] potentially due to its neuropsychiatric effects including euphoria and axiolysis [30]. Cannabis has been associated with health problems such as addiction, psychosis, and high-risk behaviors including higher alcohol consumption [31]. It has also been suggested that Cannabis consumption is harmful to periodontal tissues; however, current evidence regarding the effect of Cannabis on oral health is in general sparse and inconclusive [32]. Although both THC and CBD bind to cannabinoid receptors in the human brain and body, they produce different effects; CBD (a non-psychoactive component) has been reported to offer a wide range of health benefits including a decrease in inflammation, chronic pain, and anxiety [33]. Thus, the use of CBD and hemp oils is increasing in popularity due to medical benefits without the psychoactive effects of marijuana (low THC content) [33]. In this regard, it is pertinent to consider the ratio of THC/CBD when evaluating systemic and oral effects of vaping marijuana products. Furthermore, since e-cigarettes can be used for vaping both nicotine and cannabis compounds (THC and CBD), the co-occurring use of nicotine and cannabis is common among individuals who vape both substances [34].

Orthodontic tooth movement (OTM) depends on an active process of bone remodeling, during which alveolar bone is resorbed in the pressure side of the periodontal ligament while bone apposition occurs in the tension side [35]. This force-induced aseptic inflammation is regulated by key factors such as osteoprotegerin, prostaglandins, and cytokines [36]. Inflammation control is pertinent during OTM to avoid side effects such as alveolar bone loss, root resorption, and periodontal tissue destruction [36]. The authors of the present editorial speculate that e-cigarette vaping of nicotine, cooling agents, and/or cannabis products might alternate the inflammatory response to orthodontically induced tooth movement. Two systematic reviews have shown that the influence of nicotine on OTM has solely been examined in animal studies (mainly rats) and concluded that nicotine increases the rate of OTM by increasing alveolar bone loss and root resorption (Table 1) [1,37]. In addition, the influence of cannabis on OTM has only been examined in an experimental study in rats, which showed that THC attenuates OTM by decreasing bone resorption (Table 2) [26]. These limited experimental findings highlight the need of well-designed clinical studies to assess the influence of vaping nicotine/synthetic nicotine, cooling agents/coolants, THC/CBD, or both on OTM and periodontal tissues of patients (especially adolescents and young adults) undergoing OT with fixed appliances and/or clear aligners. Such studies will help identify whether vaping is a risk factor for periodontal diseases and bone loss during orthodontically induced tooth movement and will highlight the potential need of patient counseling for vaping cessation prior to the initiation of OT.

In summary, it is well known that the control of oral/periodontal inflammation and periodontal tissue remodeling is pertinent during OTM to prevent side effects such as alveolar bone loss and root resorption. Nicotine has been shown to increase alveolar bone loss during experimental OTM in rats potentially by impairing angiogenesis and collagen synthesis and by promoting oral inflammation and bone resorption. In addition, THC has been shown to attenuate OTM by decreasing bone resorption in rats. Overall, there is a lack of clinical and experimental studies assessing the effects of vaping nicotine, menthol or mint, VG, PG, nontraditional flavoring chemicals, and CBD and/or THC on the periodontal tissues and orthodontically induced tooth movement. The authors of the present Editorial speculate that the aforementioned vaping compounds might influence OTM and periodontal health by altering angiogenesis, wound healing, bone remodeling, and oral inflammation during the course of OT (Figure 1). Moreover, vaping these products might alter the oral microflora, further promoting periodontal and oral diseases particularly in patients undergoing OT who often find oral health maintenance challenging. In this respect, experimental studies should be conducted by examining the individual effects (and critical dosages) of these chemicals/substances on OTM and oral/periodontal health as well as their potential synergistic mechanisms. Finally, clinical studies are warranted to understand the increasing impact of vaping on the oral health of individuals undergoing OT. From a clinical perspective, it is crucial to understand the impact of rising vaping on the planned outcomes and prognosis of OT, long-term retention, and stability, as well as treatment duration and optimal frequency of orthodontic appliances activation (or frequency of tray changes in the case of clear aligner therapy). From an ethical standpoint, such studies will also highlight the potential need of patient education regarding the deleterious effects of vaping on general health and OTM and will help develop community-based programs to support vaping cessation, particularly in adolescents and young adults undergoing OT.

## Figures and Tables

**Figure 1:**
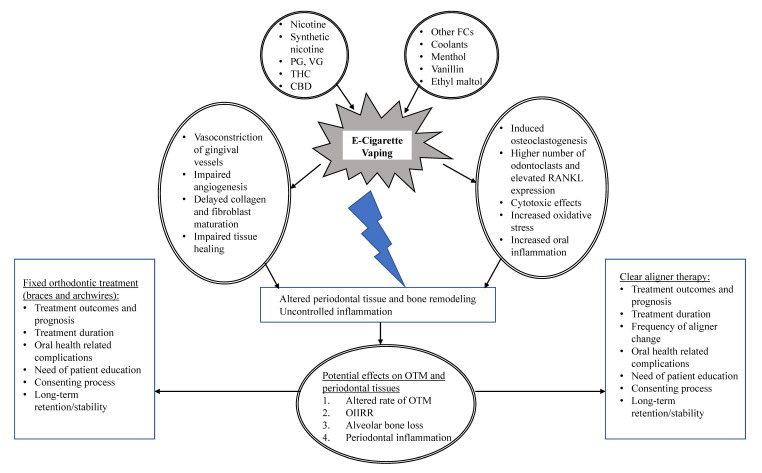
E-cigarette vaping as a risk factor for potential complications during orthodontically-induced tooth movement. Possible biological mechanisms and clinical considerations. Abbreviations: VG, vegetable glycerin; PG, propylene glycol; CBD, cannabidiol; THC, tetrahydrocannabinol; FC, flavoring chemicals; RANKL, receptor activator of nuclear factor kappa-Β ligand, OTM, orthodontic tooth movement; OIIRR, orthodontically-induced inflammatory root resorption.

**Table 1:** Studies evaluating the effect of nicotine administration and/or vaping nicotine on the rate and biology of orthodontically induced tooth movement and periodontal tissue response. OTM, orthodontic tooth movement; OIIRR, orthodontically induced inflammatory root resorption.

Effect of Nicotine Administration on OTM			Effect of Vaping Nicotine on OTM		
Reference	Study Design	Influence on OTM and Periodontal Tissue Response Compared with Control Group	Reference	Study Design	Influence on OTM and Periodontal Tissue Response Compared with Control Group
Experimental studies on animal models					
Sodagar et al. [38]	Experimental study on rats	Increased OTM and bone resorption	**No studies in indexed literature**		
Shintcovsk et al. [39]	Experimental study on rats	Reduced angiogenesis and delayed collagen maturation			
Kirschneck et al. [40]	Experimental study on rats	Increased bone loss			
Li et al. [41]	Experimental study on rats	Increased odontoclastogenesis and OIIRR			
Bakathir et al. [42]	Experimental study on rats	Increased OTM and unbalanced bone remodeling			
Kirshneck et al. [43]	Experimental study on rats	Increased OTM, bone loss and OIIRR			
Araujo et al. [44]	Experimental study on rats	No significant difference in the rate of OTM, altered collagen maturation			
Ferreira et al. [45]	Experimental study on rats	No significant difference on OTM			
Ullrich et al. [46]	Experimental study on rats	Increased OIIRR and osteoclastogenesis			
Lee et al. [47]	Experimental study on rats	No significant difference on OTM			
Clinical studies					
**No studies in indexed literature**					

**Table 2:** Studies evaluating the effects of marijuana product administration and/or marijuana vaping on the rate and biology of orthodontically-induced tooth movement and periodontal tissue response. OTM, orthodontic tooth movement.

Effect of Marijuana Product Administration on OTM			Effect of Vaping Marijuana on OTM		
Reference	Study Design	Influence on OTM and Periodontal Tissue Response Compared with Control Group	Reference	Study Design	Influence on OTM and Periodontal Tissue Response Compared with Control Group
Experimental studies on animal models					
Klein et al. [26]	Experimental study on rats	Dronabinol attenuates OTM by decreasing bone resorption	**No studies in indexed literature**		
Clinical studies					
**No studies in indexed literature**					
